# High-Intensity Interval Training Decreases Resting Urinary Hypoxanthine Concentration in Young Active Men—A Metabolomic Approach

**DOI:** 10.3390/metabo9070137

**Published:** 2019-07-10

**Authors:** Sina Kistner, Manuela J. Rist, Ralf Krüger, Maik Döring, Sascha Schlechtweg, Achim Bub

**Affiliations:** 1 Institute of Sports and Sports Science, Karlsruhe Institute of Technology, 76131 Karlsruhe, Germany; 2 Department of Physiology and Biochemistry of Nutrition, Max Rubner-Institut, 76131 Karlsruhe, Germany; 3 Department of Sport and Exercise Science, University of Stuttgart, 70174 Stuttgart, Germany

**Keywords:** metabolomics, urinary metabolome, hypoxanthine, high-intensity interval training, NMR spectroscopy, LC-MS

## Abstract

High-intensity interval training (HIIT) is known to improve performance and skeletal muscle energy metabolism. However, whether the body’s adaptation to an exhausting short-term HIIT is reflected in the resting human metabolome has not been examined so far. Therefore, a randomized controlled intervention study was performed to investigate the effect of a ten-day HIIT on the resting urinary metabolome of young active men. Fasting spot urine was collected before (−1 day) and after (+1 day; +4 days) the training intervention and 65 urinary metabolites were identified by liquid chromatography-mass spectrometry (LC-MS) and nuclear magnetic resonance (NMR) spectroscopy. Metabolite concentrations were normalized to urinary creatinine and subjected to univariate statistical analysis. One day after HIIT, no overall change in resting urinary metabolome, except a significant difference with decreasing means in urinary hypoxanthine concentration, was documented in the experimental group. As hypoxanthine is related to purine degradation, lower resting urinary hypoxanthine levels may indicate a training-induced adaptation in purine nucleotide metabolism.

## 1. Introduction

Over the past decade, research interest in HIIT as a time-efficient method for inducing health benefits has greatly increased [1]. A growing body of evidence supports the potential of HIIT to cause similar or even superior improvements in skeletal muscle energy metabolism and cardiometabolic health as compared to moderate-intensity continuous exercise [2,3]. In order to allow physicians and sports scientists to recommend more specific exercise programs or training strategies, it is crucial to know more about the cellular mechanisms and metabolic alterations underlying the whole-body and skeletal muscle adaptation to HIIT. On a molecular level, there is already strong evidence that higher exercise intensities lead to a stronger expression of peroxisome proliferator-activated receptor gamma coactivator (PGC)-1α [4,5], which is regarded as a major regulator of mitochondrial biogenesis in human skeletal muscle [6]. Given the positive effects that seem to be associated with an increase in muscle PGC-1α (e.g., an increase in skeletal muscle oxidative capacity or a greater reliance on fat oxidation [7]) the elevated activity of PGC-1α following HIIT underpins the potential of HIIT to stimulate long-term metabolic changes that promote widespread health benefits [2,8]. However, until recently, the majority of studies have focused on the investigation of a small number of key molecules or selected metabolites in order to elucidate metabolic adaptations to HIIT [5,9,10,11]. Since several biochemical pathways are interacting with each other, capturing a broader view of changes in metabolism is required.

The emerging metabolomics method offers a more comprehensive approach to simultaneously analyze a wide range of metabolites. Metabolomics facilitates the systematic identification and quantification of a large number of metabolites, defined as low-molecular weight compounds, by using analytical techniques like NMR spectroscopy, LC-MS or gas chromatography-mass spectrometry (GC-MS) [12]. The complete set of endogenous metabolites like sugars, amino acids, amines, alcohols, steroids or nucleosides as well as metabolites of exogenous origin in a given biological system—e.g., biofluids like blood or urine—represent the so-called “metabolome”. It is determined by age and sex [13] and can be influenced by external factors like nutrition [14] or physical activity [15,16]. Assessing the metabolome as comprehensively as possible, metabolomics can provide valuable information on human metabolism and its response to physiological challenges like physical exercise [17]. Thus, metabolomics contributes to the identification of exercise-responsive biomarkers, which could act as predictors of exercise-specific changes in metabolism. Metabolomics can also give insight into exercise-induced alterations in metabolite profiles, which are possibly associated with particular physiological processes or metabolic pathways.

Until now, a few studies have investigated the HIIT-induced modifications of the human metabolome by using metabolomics. The majority of studies have analyzed the acute metabolic effects of a single HIIT or sprint interval session [18,19,20,21,22,23,24,25], documenting higher concentrations of lactate and pyruvate, which reflect a higher reliance on anaerobic energy production [18,20,21,23,24,25], and/or hypoxanthine, which is known to indicate a high rate of adenosine triphosphate (ATP) turnover [18,23,24]. However, the middle- and long-term effects of HIIT on the metabolome have rarely been examined so far. The limitations of existing studies are that training interventions were partly restricted to overweight individuals [26,27] and performed with a moderate frequency [19,26,27]. Furthermore, metabolic alterations were only investigated in blood by using either NMR [19] or multi-segment injection-capillary electrophoresis-mass spectrometry (MSI-CE-MS) [26,27]. Nevertheless, these studies provide first evidence that high-intensity intermittent training is able to induce adaptive metabolic responses. Amongst others, this was shown by lower levels of lactate and pyruvate in both resting and post-exercise serum samples [19] or an attenuated exercise-induced increase in plasma hypoxanthine levels after HIIT training [27], hinting at a lower energetic stress in the trained status.

However, to the best of our knowledge, no other study has investigated to what extent the body’s adaptation to an extremely demanding HIIT intervention is reflected in the urinary metabolome. Especially in elite sports, athletes are often confronted with exhausting training protocols consisting of high-intensity exercise blocks, provoking a higher adaptation in performance and an improved fatigue resistance [1,28]. Therefore, the purpose of this study was to investigate the effect of a ten-day high-intensity interval training, which is comparable to a pre-competition preparation phase in a periodized training schedule, on the resting urinary metabolome of young active men. This is the first randomized controlled intervention study using a combined NMR- and LC-MS-based metabolomic approach to assess changes in urinary metabolite profiles, thereby aiming to identify possible biomarkers for either training overload or training adaptation. The detected urinary metabolites represent a broad spectrum of organic compounds found in the human urine metabolome [29], ranging from amino acids to alcohols, keto acids to purine derivatives and methylated amines. Urine as a biological matrix was chosen as it is easily accessible, stable and under no homeostatic regulation like other biofluids [29,30]. As the urinary metabolome was captured in the resting state—i.e., in the morning before and after the ten-day HIIT period—this study was particularly suitable to analyze if human metabolism is able to restore its disturbed homeostasis, which could be documented in response to acute HIIT [18,19,20,21,22,23,24] and can also be assumed due to an exhausting, daily performed HIIT like in this study.

## 2. Results

Descriptive characteristics of the participants and training protocols are summarized by means and standard deviations in Table 1. There were no significant differences between the experimental group (EG) and the control group (CG) with regard to age, anthropometric parameters and physical fitness at baseline. Parameters used to create individual training protocols are only presented for the EG. During daily training sessions, subjects of the EG performed eight cycling bursts at an average intensity of 348 watt (=P_max_) and for a mean duration of 97 s (=60% T_max_), interrupted by a rest period until heart rate decreased to an average of 124 beats per minute (=65% of HR_max_).

### 2.1. Training Parameters

**Figure A1:**
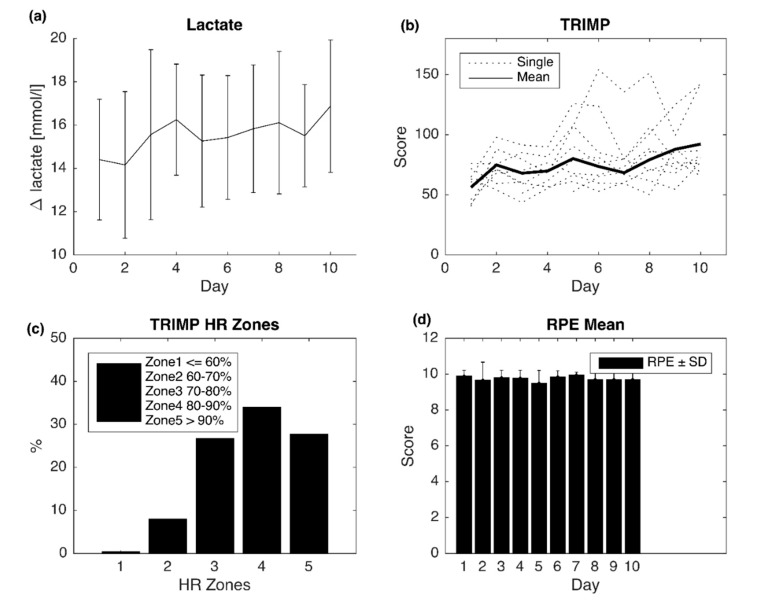
Training Parameters (**a**) Post-exercise increase in blood lactate concentration (mean ± SD); (**b**) Mean and single TRIMP score during training intervention; (**c**) Heart rate zone scaling (% of time spent in each HR zone); (**d**) RPE score (mean ± SD); HR: heart rate, RPE: rating of perceived exertion, SD: standard deviation, TRIMP: Training Impulse.

Training parameters demonstrate a high intensity of the daily training (see Figure A1). In the EG, an increase in blood lactate concentration was documented after each training session and the post-exercise increase in lactate was 14.2 ± 2.7 mmol/L on the first training day and 16.3 ± 2.7 mmol/L on the last training day. The average training impulse (TRIMP), which is considered as an objective tool to assess physical effort, increased continuously over the intervention period (from 56.2 ± 13.4 (day 1) to 96.6 ± 30.0 (day 10)). The heart rate zone scaling shows that the majority of the EG performed in heart rate zones 3–5, which means that they largely exercised at 70–100% of HR_max_. The rating of perceived exertion (RPE), which is a subjective tool to evaluate exercise intensity, was continuously at a high level in the EG and did not differ between the training days (mean ± SD (day 1–10): 9.6 ± 0.5; inter-subject mean ± SD: 9.6 ± 0.2, values are scores in the RPE scale). Regarding the values of the multidimensional questionnaire for subjective well-being (MDBF, German: Mehrdimensionaler Befindlichkeitsfragebogen), the mean score from the EG immediately decreased after the first training session and remained lower than pre-training during the whole training intervention. In the CG, no changes in MDBF values were observed during the study period.

### 2.2. Urinary Metabolites

A total of 65 urinary metabolites were identified and quantified by NMR spectroscopy and LC-MS, including creatinine for standardization purposes. Normalization of urinary metabolite concentrations to urinary creatinine was conducted with creatinine values obtained by NMR. The NMR signal intensity of creatinine and creatinine concentrations measured with a photometric assay were highly correlated (Pearson’s correlation, r = 0.99, *p* < 0.001; see Supplementary Table S1), verifying the validity of the quantitative NMR data.

**Figure 1:**
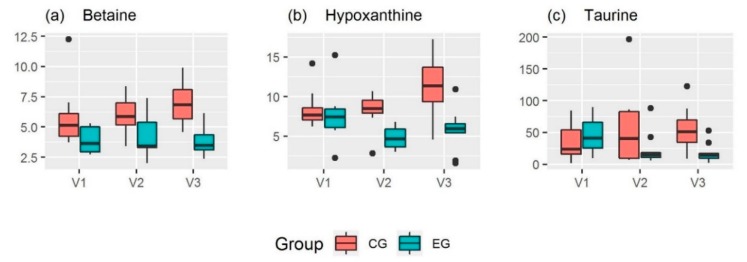
Boxplots for top-ranked metabolites (concentrations shown as mmol/mol creatinine). (**a**) Betaine (EG: n = 9, CG: n = 7); (**b**) Hypoxanthine (EG: n = 10, CG: n = 8); (**c**) Taurine (EG: n = 10, CG: n = 7); CG: Control Group, EG: Experimental Group, p: *p*-value, V1: Visit 1 (day before the training started), V2: Visit 2 (day after the the last training session), V3: Visit 3 (after four days of recovery); metabolites were chosen as top-ranked if at least one *p*-value was lower than 0.01.

Spot urine samples were collected one day before intervention started (Visit 1, V1) as well as one and four days after intervention (Visit 2, V2 and Visit 3, V3, respectively). The normalized urinary metabolite concentrations before and after the 10-day high-intensity interval training are presented in Table A1, separated for EG and CG, and as boxplots in the Supplementary Figure S1. Boxplots of top-ranked metabolites are shown in Figure 1.

Regarding pre-training urinary metabolite concentrations, no statistically significant differences between the EG and the CG could be documented. At V2, hypoxanthine (*p* = 0.0210) showed significantly different urinary concentrations between the EG and the CG, whereas at V3, urinary concentrations of betaine (*p* = 0.0055), hypoxanthine (*p* = 0.0006) and isoleucine (*p* = 0.0285) significantly differed between both groups. With respect to metabolite alterations within the EG, three metabolites significantly changed over time. Firstly, urinary hypoxanthine concentration showed a significant difference with decreasing means between V1 and V2 (*p* = 0.0270). Secondly, urinary taurine concentration significantly differed with decreasing means between V1 and V3 (*p* = 0.0031), as well as urinary asymmetric dimethylarginine (ADMA) concentration (*p* = 0.0380). Within the CG, urinary concentration of hypoxanthine significantly changed with increasing means between V1 and V3 (*p* = 0.0437) and V2 and V3 (*p* = 0.0442). Furthermore, urinary concentrations of citrulline (*p* = 0.0205), N-methylarginine (*p* = 0.0134), methylsuccinate (*p* = 0.0357) and urea (*p* = 0.0159) showed a significant difference with increasing means between V1 and V3 while no significant changes in urinary metabolites were demonstrated between V1 and V2.

## 3. Discussion

The purpose of this study was to investigate the effect of a ten-day HIIT on the resting urinary metabolome of young active men, thus analyzing if human metabolism is able to recuperate from an extremely demanding exercise intervention. The main finding of this study is that no overall change in resting urinary metabolome was observed in the EG, except for a significant difference with decreasing means in urinary hypoxanthine concentration between V1 and V2 and a significant difference with decreasing means in urinary taurine and ADMA concentrations between V1 and V3. Assuming that acute alterations in urinary metabolite concentrations have occurred in response to the daily HIIT sessions—as shown by previous studies with similar high-intensity exercise protocols but with immediate sample taking [18,19,20,24]—the almost unchanged urinary metabolome indicates that metabolism was largely able to restore its disturbed homeostasis in the given time frame of one or four recovery days, respectively. Although urine as a biological matrix has not been widely used in exercise metabolomics studies, the ability of urine to reflect acute exercise-induced metabolic changes occurring in muscle was recently demonstrated [18,20,24]. Consequently, the fact that no longer-lasting, overall metabolic alterations in urine have been documented in our study leads to the assumption that skeletal muscle metabolism had already regenerated from acute disturbances due to HIIT intervention. Acute metabolic changes in response to a single HIIT or comparable sprint interval training session are particularly characterized by elevations in plasma or urinary lactate [18,20,21,23,31], confirming that a high percentage of the energy required in muscle is provided by the anaerobic energy system [32]. Furthermore, acute elevations in plasma or urinary hypoxanthine were detected [18,20,24,31]. The purine derivative hypoxanthine represents the final ATP breakdown product in muscle and was proposed as a metabolic indicator of exercise-induced energetic stress [33,34]. If during high-intensive exercise the ATP degradation rate in muscle is higher than the ATP resynthesis rate, accumulated hypoxanthine can be partly reconverted to inosine monophosphate (IMP) by the purine salvage enzyme hypoxanthine guanine phosphoribosyltransferase (HGPRT), thus contributing to ATP restoration, or it leaks out to the blood stream where a further degradation to uric acid or a direct elimination from body via urine occurs [33,35,36,37]. An elevated urinary elimination of hypoxanthine and its downstream metabolite, uric acid, are seen as the endpoint of purine metabolism, representing an indirect marker of the exercise-induced net ATP loss from muscle to plasma [38,39].

However, despite the high intensity of the training protocol used in our study, we did not observe an increase in urinary hypoxanthine concentration one day after the last training session. This observation is in line with results of Stathis et al., which showed that urinary hypoxanthine excretion rate was only significantly elevated in the first 2 h of recovery, whereas 2 to 24 h post-exercise no increased urinary hypoxanthine concentration was documented any more [38]. As even lower post-training urinary hypoxanthine levels were demonstrated in our study (see Table A1 and Figure 1b), the question arises if adaptations in purine metabolism as a response to the exhausting HIIT have occurred, resulting in a reduced muscular hypoxanthine loss into blood even in resting conditions and finally leading to a decreased delivery of hypoxanthine to the kidneys and consequently to a lower hypoxanthine excretion via urine. Actually, this is the first study showing a decrease in resting urinary hypoxanthine levels after a short-term, but high-intensive interval training. A previous study focusing on training-induced alterations in purine metabolism observed changes in resting as well as post-exercise plasma hypoxanthine concentrations in elite athletes during a one-year training cycle [40,41]. Indeed, it was shown that resting (and post-exercise) plasma hypoxanthine decreased from the general to the competition phase, along with the increasing contribution of high-intensity anaerobic training loads, whereas in the detraining phase, resting (and post-exercise) plasma hypoxanthine concentration rose and returned to high levels [40,41]. According to Zielinski et al., plasma hypoxanthine concentrations at rest and after standard exercise can be seen as sensitive metabolic indicators of training status, providing valuable information about either training adaptation or overtraining [41]. In contrast to this, another study showed that a seven-day intermittent sprint training on a bicycle ergometer did not change resting urinary hypoxanthine concentration in active men [42]. As compared to our study, subjects were untrained and performed a slightly shorter and less intensive training intervention, which could explain the discrepant results. However, several studies documented an attenuated exercise-induced accumulation of plasma or urinary hypoxanthine when an acute maximal activity was performed after repeated high-intensity training compared to pre-training, suggesting a decrease in muscular purine nucleotide loss due to an improved balance between the rates of ATP degradation and its resynthesis [27,42,43,44,45,46]. Hellsten-Westing et al. demonstrated that the attenuation in hypoxanthine efflux after a six-week HIIT in habitually active men was associated with a training-induced, elevated activity of the purine salvage enzyme HGPRT in muscle [45]. As HGPRT catalyzes the reconversion of hypoxanthine to IMP [35], its intensified activity following HIIT was interpreted as a training-induced adaptation to enhance the conservation of muscle purine nucleotides [45]. Such an adaptation seems advantageous, because a lower loss of purines results in a lower reliance on the comparably slow and metabolically expensive replacement of adenine nucleotides by the purine de novo synthesis pathway [42].

Unfortunately, neither muscular HGPRT nor acute post-exercise alterations in hypoxanthine concentration have been analyzed in the present study. Therefore, it only can be speculated that the decreased resting urinary hypoxanthine levels are evidence of an increased ability of the body to conserve or restore the purine nucleotide pool. A previous study has shown a decrease in resting muscular adenine nucleotide levels after one week of high-intensity intermittent training [46]. Although not analyzed in the present study, a depletion of muscular adenine nucleotide content in the course of the 10-day HIIT is also presumable due to the high intensity and frequency of training. As one possible metabolic adaptation to the short-term HIIT, an elevated HGPRT activity in skeletal muscle could be suggested. However, if muscular HGPRT activity actually increased after ten days of HIIT, it is still questionable to what extent a more efficient salvage in muscle is likely to explain the decrease in urinary hypoxanthine excretion at rest. Indeed, urinary hypoxanthine levels not only depend on muscular hypoxanthine release, but also on its uptake by red blood cells [47] or the liver and its further degradation to uric acid [33,34]. Thus, it cannot be ruled out that an increased hypoxanthine uptake by other, non-muscle tissues underlay the observed decrease in resting urinary hypoxanthine concentration. As recently shown, not only HGPRT activity in skeletal muscle but also HGPRT activity in erythrocytes is exercise- and training-dependent [48,49,50]. Dudzinska et al. demonstrated that trained subjects had a significantly higher HGPRT activity in erythrocytes at rest [48], which might contribute to the reduction of resting plasma as well as urinary hypoxanthine levels by utilizing more hypoxanthine for IMP formation.

Since resting urinary hypoxanthine concentration in the EG was still lower at V3 than pre-training and not significantly different from V2 (see Table A1 and Figure 1b), the possibility exists that the performed training induced an increase in purine salvage efficiency, however it is still unclear in which tissues this adaptation occurred. Providing detailed information about underlying molecular mechanisms of adaptation to HIIT is out of the scope of this study. It is obvious that subjects of the EG were exposed to a remarkable metabolic and psychological stress, as indicated by an increase in post-exercise blood lactate and a decrease in MDBF scores during intervention, which reflect subjective well-being [51]. Nevertheless, adaptations to the exhaustive HIIT intervention were also demonstrated. The TRIMP as a marker of training load continuously increased over the 10-day intervention period in the EG (see Figure A1b), showing that participants were able to realize a higher physical effort in the course of the intervention. Regarding this and the fact that urinary hypoxanthine levels in the EG significantly changed post-training, being significantly different from urinary hypoxanthine levels in the CG at V2 and V3, alterations in purine metabolism could provide one possible mechanism of the body’s adaptation to HIIT training. Unfortunately, we are not able to explain the significant differences with increasing means in urinary hypoxanthine levels in the CG between V1 and V3 and V2 and V3, respectively. It is obvious that the CG showed a higher variation in urinary hypoxanthine concentrations at V3 when compared with V1 and V2 (see Table A1 and Figure 1b). We assume that the documented differences are due to a natural variation, which could not be controlled in our study.

The biological relevance of the change in resting urinary taurine levels between V1 and V3 in the EG (see Table A1 and Figure 1c) has also to be interpreted with caution, since it is known that the urinary excretion of this semi-essential amino acid varies along with dietary taurine intake [52]. Although urine samples were collected after an overnight fast and after the intake of a standardized evening meal, it cannot be excluded that participants consumed different amounts of taurine-containing food like meat, fish, shellfish or energy drinks [52,53] in the days before urine collection. However, regarding the decrease in urinary taurine within the EG, it could be hypothesized that more taurine was utilized during HIIT intervention. Taurine is highly concentrated in the muscles [54] and involved in cell volume regulation, calcium homeostasis, membrane stabilization and antioxidant activities [52], all important with respect to physical exercise. In a previous study, it was postulated that taurine is released by contracting muscles during exercise due to osmoregulatory processes, thus leading to an acute increase in plasma taurine and, when individuals were sufficiently hydrated, a higher urinary taurine clearance [55]. As taurine cannot be metabolized by humans, it is either excreted via urine or faeces [52]. To our knowledge, there is a lack of studies investigating the effect of HIIT on taurine metabolism. Another previous study analyzed the effect of endurance exercise on urinary taurine excretion. They showed that urinary taurine immediately increased post-exercise, whereas after 24 h, the urinary taurine excretion declined to slightly lower levels than baseline [56]. Assuming that in our study muscular taurine also was acutely released during HIIT intervention, it can be speculated that the lower urinary taurine levels at V3 might be a result of “restoring” the pre-exercise muscular taurine content. Since the reabsorption of taurine in the kidneys is variable, ranging from 40% to 99% [57], the possibility exists that in the regeneration phase more taurine was retained in the body, being reflected in a lower urinary taurine excretion. Unfortunately, physiological processes underlying the changes in urinary taurine in the EG remain to be elucidated.

Similar to urinary taurine levels, we documented a decrease in urinary levels of ADMA between V1 and V3 in the EG. ADMA is endogenously produced from the turnover of arginine-methylated proteins [58] and can either be metabolized to citrulline and dimethylamine or excreted via urine [58]. As ADMA functions as an inhibitor of nitric oxide synthase, high plasma ADMA levels are considered as a cardiovascular risk factor [59] and an increased urinary excretion seems to be one of the main mechanisms lowering ADMA plasma levels [60]. Recently, it was shown that physical exercise decreases ADMA levels in plasma [61]. However, the effect of exercise on urinary ADMA excretion in healthy individuals has rarely been examined so far. Despite the statistically significant change in urinary ADMA between V1 and V3 in our study, the absolute difference was marginal (see Table A1 and Supplementary Figure S1). Regarding this and the fact that we did not observe a significant difference between V1 and V2 within the EG, we assume that the change in urinary ADMA levels has no biological relevance with respect to HIIT intervention.

The significant differences with increasing means in urinary concentrations of citrulline, N-methylarginine, methylsuccinate and urea between V1 and V3 within the CG can hardly be explained and might be due to uncontrolled variation. For example, the documented increase in urinary urea levels could likely be an effect of diet—i.e., of an increased protein intake—as urea represents the terminal product of protein catabolism, being eliminated in urine [62].

Equally, we hypothesize that the difference between groups in urinary levels of isoleucine at V3 can be seen as an evidence of natural variation in the CG, leading to a higher isoleucine excretion at V3 when compared with the EG. Differences between groups in urinary betaine concentration at V3 could also be due to uncontrolled variation in the CG. However, as betaine functions as an organic osmolyte and methyl donor [63], its importance for exercise performance has recently been discussed [64]. With regard to this and the fact that we could observe a slight, but not significant increase of urinary betaine levels in the CG (see Figure 1a), it can be speculated that the CG, which had to refrain from their usual daily training during the study period, demonstrated a minor use of betaine for exercise-related physiological processes, thus leading to a higher urinary betaine excretion in the inactive state. However, as the urinary betaine levels already differed slightly at V1 between both groups, it could be also suggested that diet might account for the higher urinary betaine excretion in the CG. Differences in urinary betaine might be explained by a different dietary intake of betaine-containing food like wheat, shellfish or spinach [65,66].

A clear limitation of the present study is that the participants of the CG were instructed to refrain from heavy exercise, however, their actual physical activity behavior was not recorded. Similarly, the daily diet of study participants was not controlled. This has to be taken into account when interpreting the results, as it is known that the urinary excretion of some metabolites can be affected by diet. Another limitation of our study is that we conducted a targeted metabolomic approach, which was limited to a specific number of known compounds, and the lack of a comparative metabolomic analysis in blood, which would have been of great interest in order to facilitate the interpretation of documented metabolite alterations. Furthermore, as the present study was a follow up investigation of a study focusing on an univariate and less varying endpoint, there is a lack of statistical power regarding the analyses of urinary metabolites which show a comparatively higher biological variance. Therefore, the obtained results need to be evaluated cautiously and can only be interpreted for each single metabolite. As expected with respect to the moderate sample size, the observed changes in the metabolite concentrations are marginal when considering multiple hypotheses, which take all metabolites simultaneously into account. Consequently, the present study has to be considered as a pilot study, providing first hints about the possible effects of HIIT on urinary metabolites. More studies have to be done to confirm the observed HIIT-induced metabolic changes and to extend the obtained results to a broader population, i.e., not only to young, trained men but also to untrained individuals and females. With regard to future studies, it would be appropriate to investigate the effects of a short-term HIIT not only on resting urinary as well as blood metabolite levels but also on acute post-exercise metabolic changes in the course of intervention. Thus, a more reliable conclusion about HIIT-induced adaptations in metabolism could be drawn.

## 4. Materials and Methods

### 4.1. Subjects and Study Design

Twenty healthy men volunteered to take part in this study. All participants were regularly active, competition-experienced and participated in training at least three times a week. Further inclusion criteria were age between 20 and 50 years and BMI ≤ 30 kg/m^2^. Participants were excluded if they used any medication, or if they suffered from musculoskeletal injuries within the preceding twelve months or from chronic diseases. The study consisted of a randomized, controlled trial in which the participants were firstly subjected to standardized preliminary tests. Participants were then randomly assigned to either experimental group (EG) or control group (CG) balanced to their age and training status. Subjects of the EG took part in a ten-day HIIT intervention whereas subjects of the CG were told to refrain from heavy exercise during the intervention period. In total, two subjects of the CG dropped out of the study due to illness or technical measurement errors in blood counts, respectively. The study was approved by the ethics committee of the State Medical Chamber of Baden-Württemberg, Stuttgart, Germany (DRKS-ID: DRKS00010841) and was conducted in accordance with the declaration of Helsinki. Written informed consent was obtained from all participants prior to entering the study.

### 4.2. Preliminary Testing

Before the onset of the study, participants were subjected to a preliminary examination that included anthropometric measurements (body weight (in kg), body height (in cm) and length of the lower limbs (in cm)) and the completion of a medical history form. BMI was calculated by dividing the body weight (in kg) by the height (in meters) squared.

In order to establish an individual training protocol and as a criteria for the randomization process, maximal oxygen consumption (VO_2max_) was measured using an incremental protocol (see Laursen et al. [67]) on a bicycle ergometer (Excalibur Sport Ergometer, Lode B.V., Groningen, Netherlands). Briefly, each participant pedaled at a self-selected speed for five minutes, workload was then augmented to 100 watt and finally increased by 15 watt every 30 s until volitional exhaustion, i.e., when a pedal frequency of 60 revolutions per minute (rpm) could no longer be maintained. Respiratory gas exchange was measured breath-by-breath and heart rate was recorded. The incremental test was considered valid when the following criteria were fulfilled: (1) an initial linear increase and a subsequent plateau in oxygen consumption, with an increment of less than 2 mL/(kg/min) between the two final measurements (despite the increase in workload), (2) the achievement of 90% of the age-predicted maximum heart rate (HR_max_), (3) a respiratory exchange ratio >1.10. Endpoints of the incremental test were VO_2max_ and maximal power (P_max_), which was defined as the lowest power output at maximal oxygen consumption.

One day after the incremental test, subjects of the EG performed a sprint cycling test (see Laursen et al. [67]) in order to determine the duration of individual training protocols. Each participant pedaled at a self-selected speed for five minutes, before workload was augmented to predetermined P_max_ until exhaustion, i.e., when pedal frequency fell below 60 rpm. Endpoint of this test was T_max_, which was defined as the time the individuals performed at P_max_. Based on the pre-tests, individual training protocols were created (Table 2), which consisted of eight sets at the intensity P_max_, a duration of 60% T_max_ and a relative recovery period defined by the time needed until heart rate decreased to 65% of HR_max_ measured in the incremental test.

### 4.3. Experimental Protocol

The training protocol was completed daily for ten days in a row by the subjects of the EG. After a 5-min warm-up at 75 watt, participants performed their individual protocol on a bicycle ergometer (Excalibur Sport Ergometer, Lode B.V., Groningen, The Netherlands). Pre-training (after warm-up) and post-training capillary blood was drawn to measure lactate concentrations. To assess individual physiological and psychological stress and as a control for the training process, several tests were performed by the EG. During each training session, heart rate was continuously recorded (Polar RS800CX) to calculate the training impulse (TRIMP), following the instructions from Banister et al. [68]. The TRIMP is an objective tool to assess physical effort, considering the individual heart rate responses to training and the duration of performed exercise. The TRIMP allows the evaluation of the accumulated time in different heart rate zones based on <60%, 60–70%, 70–80%, 80–90% and >90% of HR_max_, as suggested by Edwards et al. [69]. Immediately post-training, the rating of perceived exertion (RPE) was asked, following the instructions from Foster et al. [70]. The RPE represents a subjective but valid method for quantifying exercise intensity. The RPE scale goes from zero to ten, while ten stands for maximal exertion [70]. Additionally, the EG as well as the CG daily answered a multidimensional questionnaire for subjective well-being, following the instructions from Steyer et al. [51]. All participants were asked to refrain from alcohol, performance enhancing supplements and drugs during the whole intervention. Besides, they were instructed not to change their eating habits during the study period. Evening meals the days before urine collection were standardized, insofar that participants were asked to consume pasta with tomato sauce.

### 4.4. Urine Sample Collection

Fasting spot urine from all participants was collected into polypropylene collection cups the day before the training started (Visit 1, V1), the day after the last training session (Visit 2, V2) as well as after four days of recovery (Visit 3, V3). All samples were taken between 9:00 and 10:00 a.m., centrifuged at 1850× *g* at 4 °C for 10 min, separated into aliquots and stored at −80 °C until analysis.

In order to minimize the risk of bias through exercise, V1 was scheduled at least three days after the pre-tests. Additionally, participants were instructed to refrain from physical exercise 48 h before urine collection—except participants of the EG at V2.

### 4.5. Metabolomic Analyses

A combined NMR- and LC-MS-based, targeted metabolomic approach was applied to identify and quantify a broad range of metabolites, thus making it possible to assess changes in urinary metabolome following intervention. A short overview of both analytical methods will be given in the next section; more details are available in the supplement of Rist et al. [13].

#### 4.5.1. NMR

All urine samples were analysed by 1D-^1^H-NMR spectroscopy, as previously described [13]. Briefly, urine samples were centrifuged, and supernatants mixed with 10% of a buffer containing 1.5 M KH_2_PO_4_, 2 mM NaN_3_, and 0.58 mM TSP in D_2_O at pH 7.4. Samples were measured at 300 K on a Bruker 600 MHz spectrometer (AVANCE II with 1H-BBI room temperature probe (Bruker BioSpin GmbH, Rheinstetten, Germany)) equipped with a BACS sample changer. Quality control (QC) samples were prepared by pooling fasting urine samples from all participants. At least one QC sample was analysed per 24 study samples. The identification and quantitative analysis of 47 urinary metabolites, including organic acids, amino acids, amines, sugars, sugar alcohols and others, was carried out with Chenomx NMR Suite 8.1 (Chenomx, Edmonton, AB, Canada). Imprecision was generally <10% for most analytes, except for succinate. The original NMR dataset as well as technical precision are presented in the Supplementary Table S2.

#### 4.5.2. LC-MS

In addition to NMR analysis, a targeted ultra-performance liquid chromatography-tandem mass spectrometry (UPLC-MS/MS) analysis of methylated amino compounds was conducted using an Acquity H-Class UPLC coupled to a Xevo TQD triple quadrupole MS (both from Waters, Eschborn, Germany). The method was originally developed for the analysis of trimethylamine N-oxide (TMAO) and five related compounds in plasma samples (see [13,71]), but was adapted to urine and extended to 28 analytes in this study. Briefly, urine samples were diluted 25 times with eluent A (1:1 acetonitrile/aqueous 50 mM ammonium formate) and separated by an inverse acetonitrile gradient on a polar HILIC column (Acquity BEH Amide, 100 × 2.1 mm, 1.7 µm, Waters, Eschborn, Germany). Analytes were detected by positive ESI and timed MRM. Matrix-matched calibrators and controls (3 levels), dedicated isotope-labelled internal standards and standard addition were used for quantification. Imprecision was generally <15% for most analytes and levels, with few exceptions. The original LC-MS dataset as well as technical precision are presented in the Supplementary Table S3. 13 of the detected metabolites show concentrations below the lower limit of quantification (LLOQ), which was defined by the following criteria: Signal to noise ratio 10:1, imprecision and bias <30%. However, all values (including values below LLOQ) were considered for statistical calculations, since the occurrence of such values is a property of the population. Although it is known that the single values below the LLOQ are more inaccurate, such values contain valuable information for the description of the whole sample. The use of the values below the LLOQ is, with respect to the statistical properties, better than any artificial replacement. The particular LLOQs as well as the specific number of data below the LLOQ were indicated in the Supplementary Table S4.

Nine urinary metabolites (β-aminoisobutyrate, betaine, carnitine, creatine, dimethylamine, N,N-dimethylglycine, histidine, trigonelline and TMAO) were accessible by both NMR and LC-MS. Analyte concentrations obtained by NMR and LC-MS were compared by linear regression. Results are presented in the Supplementary Figure S2. In brief, the correlations were strong in all cases with R^2^ > 0.87. Absolute values did also match closely, reflected by slopes near 1 and small intercepts. The only exceptions were TMAO and dimethylamine, where significant deviations between LC-MS and NMR were observed. The reason remains unclear, but is likely related to different standardization and should be considered when comparing results from studies using different methods. Overall, we observed a high correlation between NMR and LC-MS data in our study. LC-MS analysis generally offers a higher sensitivity than NMR [72] and peak assignment and integration in NMR is sometimes critical and pH-dependent. Therefore, further data handling and statistical analysis for the named metabolites were carried out with the values obtained by LC-MS.

### 4.6. Data Handling and Statistical Analysis

Metabolite concentrations were normalized to urinary creatinine, thus controlling for variations in urine dilution. Urinary creatinine concentrations were obtained through ^1^H-NMR spectroscopy and verified by the creatinine concentrations measured using a photometric assay based on the Jaffe reaction (DetectX^®^ Urinary Creatinine Detection Kit, K002-H5, Arbor Assays, Ann Arbor, MI, USA). Differences between groups in anthropometric characteristics, fitness parameters and baseline metabolite concentrations were examined by Welch’s *t*-test. In order to investigate differences in urinary concentrations for each metabolite, a mixed effect model with treatment (EG, CG), time (V1, V2, V3) and treatment-time interaction as fixed factors were applied. To take account of the repeated measurements and varying baseline values, the random factors were modelled by a random intercept and a general correlation structure of the error terms. Tukey type contrasts were tested for by multiple testing adjusted *t*-tests. As the applied *t*-tests were considered robust to deviations from normality assumption and due to a moderate sample size, parametric statistical analysis was used. No kind of multivariate statistical analysis for the consideration of all metabolites simultaneously, like false discovery corrections, were performed due to the moderate sample size. Data are presented as mean ± standard deviation. The level of statistical significance for all analyses was set at α = 0.05. Statistical analysis was performed using SAS JMP 11.0.0. (SAS Institute Inc. 2013, Cary, NC, USA) and the software R version 3.4.2 [73] using packages nlme [74] and lsmeans [75]. Sample size can partly differ due to missing data, e.g., when technical measurement errors occurred or because measured values were excluded due to biological implausibility.

## 5. Conclusions

We investigated the effect of a ten-day HIIT on 65 urinary metabolites in young active men. To our knowledge, this was the first study using a combined NMR- and LC-MS-based metabolomic approach to assess changes in resting urinary metabolome, which possibly are related to the body’s adaptation to a HIIT protocol comparable to pre-competition training schedules. Our findings show that no overall change in resting urinary metabolome, except a decrease in urinary hypoxanthine concentration, was caused in the EG one day after the HIIT intervention. This result indicates that metabolism was able to quickly regenerate from acute metabolic disturbances due to the exhausting HIIT. However, as resting urinary hypoxanthine levels were lower and significantly different following HIIT, training-induced adaptations in purine nucleotide metabolism can be suggested. To reveal underlying mechanisms, further studies are necessary.

## Figures and Tables

**Table 1:** Descriptive characteristics of participants and training protocols.

	Total (n = 18)			EG (n = 10)			CG (n = 8)		
Age (years)	30.2	±	7.6	30.0	±	8.0	30.4	±	8.0
Height (cm)	182	±	7	181	±	7	184	±	7
Weight (kg)	79.7	±	7.6	77.1	±	8.3	82.9	±	5.5
BMI (kg/m^2^)	24.0	±	2.2	23.6	±	1.6	24.6	±	2.8
VO_2max_ (mL/(kg/min))	54.1	±	8.2	53.0	±	6.1	55.5	±	10.6
P_max_ (W)	351	±	30	348	±	31	355	±	30
HR_max_ (bpm)	182	±	11	181	±	13	185	±	8
65% HR_max_ (bpm)	-	−	-	124	±	5	-	−	-
T_max_ (s)	-	−	-	162	±	23	-	−	-
60% T_max_ (s)	-	−	-	97	±	14	-	−	-

All values in mean ± SD; BMI: body mass index, CG: control group, EG: experimental group, HR_max_: maximum heart rate, P_max_: lowest power output at VO_2max_, SD: standard deviation, T_max_: time to exhaustion at P_max_, VO_2max_: maximal oxygen consumption.

**Table 2:** Training Protocol.

Sets	Intensity	Duration	Rest
8	P_max_	60% T_max_	65% HR_max_

P_max_: lowest power output at VO_2max_, T_max_: time to exhaustion at P_max_, HR_max_: maximum heart rate.

## Supplementary Materials

The following are available online at https://www.mdpi.com/2218-1989/9/7/137/s1, Figure S1: Boxplots of urinary metabolite concentrations measured with LC-MS and NMR, Figure S2: Comparison of LC-MS and NMR metabolite data, Table S1: Comparison of creatinine data obtained by NMR and photometric assay, Table S2: Original NMR data, Table S3: Original LC-MS data, Table S4: LLOQ of LC-MS data.

## Abbreviations

ADMA	asymmetric dimethylarginine
ATP	adenosine triphosphate
BMI	body mass index
CG	control group
EG	experimental group
GC-MS	gas chromatography-mass spectrometry
HGPRT	hypoxanthine-guanine phosphoribosyltransferase
HIIT	high-intensity interval training
HR_max_	maximum heart rate
LC-MS	liquid chromatography-mass spectrometry
LLOQ	lower limit of quantification
MDBF	Mehrdimensionaler Befindlichkeitsfragebogen (*German*)
MSI-CE-MS	multi-segment injection-capillary electrophoresis-mass spectrometry
NMR	nuclear magnetic resonance
*p*	*p*-value of context-dependent test
PGC-1α	peroxisome proliferator-activated receptor gamma coactivator 1-alpha
P_max_	maximal power
QC	quality control
RPE	rating of perceived exertion
rpm	revolutions per minute
SDMA	symmetric dimethylarginine
TMAO	trimethylamine-N-oxide
T_max_	time to exhaustion at P_max_
TRIMP	training impulse
UPLC-MS/MS	ultra-performance liquid chromatography-tandem mass spectrometry
V1	visit 1 (day before the training started)
V2	visit 2 (day after the the last training session)
V3	visit 3 (after four days of recovery);
VO_2max_	maximal oxygen consumption

## Appendix A

**Table A1:** Normalized urinary metabolite concentrations at V1, V2 and V3 (Experimental Group vs. Control Group).

	EG (n = 10)									CG (n = 8)								
	V1			V2			V3			V1			V2			V3		
β-Aminoisobutyrate ^2, c)^	13	±	16	10	±	11	10	±	9	3.2	±	1.1	2.9	±	1.7	4.6	±	3.7
γ-Aminobutyrate ^2, c)^	0.10	±	0.04	0.11	±	0.05	0.10	±	0.07	0.10	±	0.07	0.13	±	0.05	0.09	±	0.03
γ-Butyrobetaine ^2, c)^	0.25	±	0.22	0.14	±	0.10	0.22	±	0.14	0.15	±	0.12	0.18	±	0.11	0.22	±	0.12
π-Methylhistidine ^2, c)^	42	±	40	45	±	40	39	±	32	19	±	27	19	±	26	10	±	8
τ-Methylhistidine ^2, c)^	18	±	3	19	±	2	18	±	2	21	±	7	18	±	4	18	±	3
1-Methylnicotinamide ^1^	3.1	±	1.1	3.8	±	1.6	3.9	±	1.3	5.9	±	3.9	4.3	±	2.6	5.7	±	2.7
2-Hydroxyisobutyrate ^1^	4.2	±	0.7	4.2	±	0.9	4.2	±	0.8	4.0	±	1.0	3.8	±	0.9	4.3	±	1.2
3-Hydroxyisovalerate ^1^	3.4	±	1.0	3.4	±	1.1	2.9	±	0.6	2.9	±	0.6	3.0	±	0.8	3.5	±	1.3
3-Indoxylsulfate ^1^	18	±	8	21	±	16	20	±	11	19	±	4	20	±	8	17	±	6
3-Methylxanthine ^1^	5.2	±	3.5	6.4	±	3.1	6.3	±	5.4	4.3	±	2.6	3.6	±	1.6	5.5	±	4.3
4-Hydroxyphenylacetate ^1^	8.7	±	8.5	8.5	±	4.0	7.9	±	5.5	6.2	±	1.6	6.9	±	2.6	6.3	±	2.5
Acetate ^1^	2.8	±	1.4	3.2	±	2.4	2.9	±	1.9	2.9	±	1.7	3.5	±	2.0	3.3	±	1.0
Acetone ^1, e)^	1.1	±	0.5	1.0	±	0.6	1.0	±	0.9	1.2	±	1.0	1.8	±	2.4	1.5	±	0.8
ADMA ^2, c)^	3.7	±	0.4 †^b^	3.6	±	0.5	3.4	±	0.4 †^b^	3.8	±	0.7	3.8	±	0.6	3.7	±	0.6
Alanine ^1^	14	±	6	15	±	6	14	±	6	14	±	6	16	±	5	13	±	5
Anserine ^2, c)^	0.18	±	0.18	0.23	±	0.23	0.13	±	0.10	0.29	±	0.59	0.12	±	0.12	0.06	±	0.02
Arginine ^2, c)^	2.1	±	0.9	1.9	±	0.5	1.7	±	0.6	1.8	±	0.5	2.7	±	2.7	2.1	±	0.6
Betaine ^2, a) c)^	3.9	±	1.1	4.3	±	1.8	3.9	±	1.3 *	6.0	±	3.0	6.0	±	1.7	7.0	±	1.9 *
Betonicine ^2, c)^	0.64	±	1.38	0.28	±	0.34	0.19	±	0.21	0.09	±	0.10	0.10	±	0.15	0.04	±	0.03
Carnitine ^2, c)^	12	±	10	10	±	9	15	±	11	10	±	10	10	±	6	15	±	5
Carnosine ^2, c)^	1.0	±	0.6	1.3	±	0.7	1.2	±	0.5	1.3	±	0.7	1.4	±	0.6	1.3	±	0.7
Choline ^2, c)^	2.0	±	1.1	1.9	±	0.9	1.6	±	0.5	1.7	±	0.7	2.3	±	1.7	1.8	±	0.3
cis-Aconitate ^1^	10	±	2	11	±	2	11	±	3	11	±	4	12	±	4	13	±	8
Citrate ^1^	200	±	130	200	±	130	190	±	100	200	±	100	200	±	70	230	±	70
Citrulline ^2, c)^	0.7	±	0.4	0.6	±	0.3	0.6	±	0.3	0.5	±	0.3 †^b^	1.3	±	1.8	1.1	±	0.7 †^b^
Creatine ^2, a) c)^	2.6	±	0.8	2.4	±	0.6	3.2	±	2.7	4.1	±	3.4	4.2	±	3.2	3.1	±	1.4
Dimethylamine ^2, c)^	23	±	7.8	21	±	2	20	±	3	21	±	2	19	±	2	22	±	3
*N*,*N*-Dimethylglycine ^2, a) c)^	2.3	±	0.8	2.7	±	1.3	2.2	±	0.9	2.5	±	1.1	2.7	±	0.7	2.8	±	0.8
Dimethylsulfone ^1^	4.2	±	3.6	3.9	±	2.8	3.9	±	3.1	3.3	±	1.4	4.5	±	4.4	4.3	±	2.2
Formate ^1^	11	±	5	13	±	9	13	±	7	13	±	5	15	±	9	16	±	5
Gluconate ^1^	24	±	8	26	±	6	24	±	5	24	±	5	23	±	7	2	±	7
Glycine ^1^	80	±	50	72	±	40	78	±	41	60	±	22	61	±	16	62	±	24
Glycolate ^1^	29	±	8	31	±	8	30	±	11	37	±	13	34	±	9	34	±	8
Guanidoacetate ^1^	13	±	4	11	±	4	11	±	5	13	±	5	14	±	6	13	±	4
Hippurate ^1^	140	±	80	170	±	110	140	±	70	250	±	130	190	±	80	170	±	110
Histidine ^2, c)^	58	±	22	59	±	21	55	±	25	60	±	28	60	±	15	57	±	12
Hypoxanthine ^1^	7.6	±	3.3 †^a^	4.8	±	1.4 * †^a^	5.8	±	2.6 *	8.5	±	2.7 †^b^	8.2	±	2.4 * †^c^	11.4	±	3.9 * †^b^ †^c^
Isoleucine ^1^	0.72	±	0.32	0.81	±	0.22	0.61	±	0.19 *	0.70	±	0.20	0.78	±	0.20	0.95	±	0.51 *
Lactate ^1^	3.6	±	0.8	3.8	±	1.2	3.8	±	1.4	3.6	±	1.1	4.3	±	1.9	3.7	±	0.8
Leucine ^1^	2.0	±	0.5	2.0	±	0.4	1.9	±	0.5	1.9	±	0.2	2.0	±	0.6	2.0	±	0.3
Mannitol ^1, c)^	8.1	±	4.8	10.4	±	4.4	10.5	±	5.7	5.3	±	5.1	6.3	±	2.5	10.2	±	8.8
Methanol ^1^	2.9	±	2.5	4.4	±	4.2	3.7	±	4.5	2.6	±	2.4	4.5	±	6.1	3.6	±	2.5
Methylamine ^1, b)^	2.9	±	1.6	2.4	±	1.2	2.3	±	1.0	2.3	±	0.8	2.4	±	0.7	2.2	±	0.7
*N*-Methylarginine ^2, c)^	0.03	±	0.03	0.02	±	0.01	0.02	±	0.02	0.01	±	0.01 †^b^	0.04	±	0.06	0.04	±	0.03 †^b^
*N*-Methylproline ^2, a) c)^	0.08	±	0.07	0.20	±	0.31	0.09	±	0.04	0.25	±	0.34	0.15	±	0.15	0.19	±	0.16
Methylsuccinate ^1^	5.4	±	1.4	6.1	±	2.3	5.7	±	1.4	6.1	±	2.4 †^b^	6.2	±	1.1	7.4	±	3.4 †^b^
Proline ^2, a) c)^	0.61	±	0.28	0.56	±	0.15	0.53	±	0.07	0.67	±	0.46	1.10	±	1.17	0.91	±	0.43
Pseudouridine ^1^	11	±	1	11	±	1	11	±	1	11	±	1	11	±	1	11	±	1
Pyruvate ^1^	2.2	±	0.8	2.2	±	0.7	2.0	±	0.5	2.0	±	0.6	2.6	±	1.3	1.8	±	0.5
Sarcosine ^2, a) c)^	0.05	±	0.02	0.05	±	0.02	0.06	±	0.05	0.09	±	0.06	0.11	±	0.11	0.07	±	0.06
SDMA ^2, c)^	33	±	4	35	±	4	33	±	3	34	±	2	31	±	1	33	±	6
Stachydrine ^2, c)^	15	±	19	28	±	30	17	±	12	17	±	16	11	±	11	12	±	11
Succinate ^1^	1.4	±	0.7	2.1	±	1.4	2.0	±	1.1	2.5	±	1.3	2.3	±	1.8	2.3	±	0.8
Tartrate ^1, b) d)^	1.8	±	1.4	2.9	±	3.0	2.1	±	1.8	1.6	±	1.1	1.9	±	1.4	1.3	±	0.5
Taurine ^1, c)^	44	±	28 †^b^	23	±	25	18	±	15 †^b^	36	±	32	61	±	68	56	±	38
Threonine ^1^	6.8	±	2.2	6.8	±	1.8	7.2	±	2.9	7.6	±	2.4	7.6	±	2.7	7.2	±	3.4
trans-Aconitate ^1^	3.2	±	0.7	3.9	±	1.4	3.5	±	0.7	3.1	±	0.6	2.9	±	1.7	3.0	±	0.8
Trigonelline ^2, c)^	15	±	15	12	±	10	14	±	11	12	±	8	11	±	7	11	±	10
Trimethylamine ^2, c)^	0.28	±	0.17	0.25	±	0.10	0.20	±	0.07	0.25	±	0.09	0.31	±	0.19	0.20	±	0.06
TMAO ^2, c)^	56	±	55	45	±	28	37	±	23	36	±	17	30	±	12	58	±	42
Tyrosine ^1^	7.4	±	2.6	7.5	±	2.4	7.1	±	2.5	8.0	±	2.7	8.6	±	3.7	8.1	±	2.6
Uracil ^1^	7.2	±	2.8	6.9	±	3.7	6.8	±	3.1	6.0	±	1.4	5.9	±	2.5	6.0	±	1.1
Urea ^1^	2400	±	1100	2700	±	1100	2300	±	800	2100	±	500 †^b^	2600	±	1400	2900	±	700 †^b^
Valine ^1^	2.3	±	0.7	2.5	±	0.6	2.4	±	0.6	2.6	±	0.4	2.6	±	0.4	2.6	±	0.5

All values in mean ± SD; urinary metabolite concentrations were normalized to urinary creatinine concentration and shown as mmol/mol creatinine; ADMA: asymmetric dimethylarginine, CG: control group, EG: experimental group, *p*: *p*-value, SDMA: symmetric dimethylarginine, TMAO: trimethylamine-N-oxide, V1: Visit 1 (day before the training started), V2: Visit 2 (day after the last training session), V3: Visit 3 (after four days of recovery); ^1^ NMR-detected, ^2^ LC-MS-detected, ^a)^ n = 9 for EG, ^b)^ n = 9 for EG only at V2, ^c)^ n = 7 for CG, ^d)^ n = 7 for CG only at V2, ^e)^ n = 7 for CG only at V3; * *p* ≤ 0.05, significant difference between EG and CG at V2 and V3, respectively; †^a^ *p* ≤ 0.05, significant difference between V1 and V2 within one group †^b^ *p* ≤ 0.05, significant difference between V1 and V3 within one group; †^c^ *p* ≤ 0.05, significant difference between V2 and V3 within one group.
