# Supplementary material for: High-Intensity Interval Training Decreases Resting Urinary Hypoxanthine Concentration in Young Active Men—A Metabolomic Approach
Source: Metabolites. 2019 Jul 10;9(7):137. doi: 10.3390/metabo9070137 (PMC6680906; doi:10.3390/metabo9070137)
Supplement: Supplementary file 1 [file metabolites-09-00137-s001.zip › Figure S1_Boxplots of urinary metabolite concentrations measured with LC-MS and NMR.pdf]

Figure S1: Boxplots of urinary metabolite concentrations measured with LC-MS and NMR.

Urinary metabolite concentrations were normalized to urinary creatinine concentrations and shown as mmol/mol creatinine.

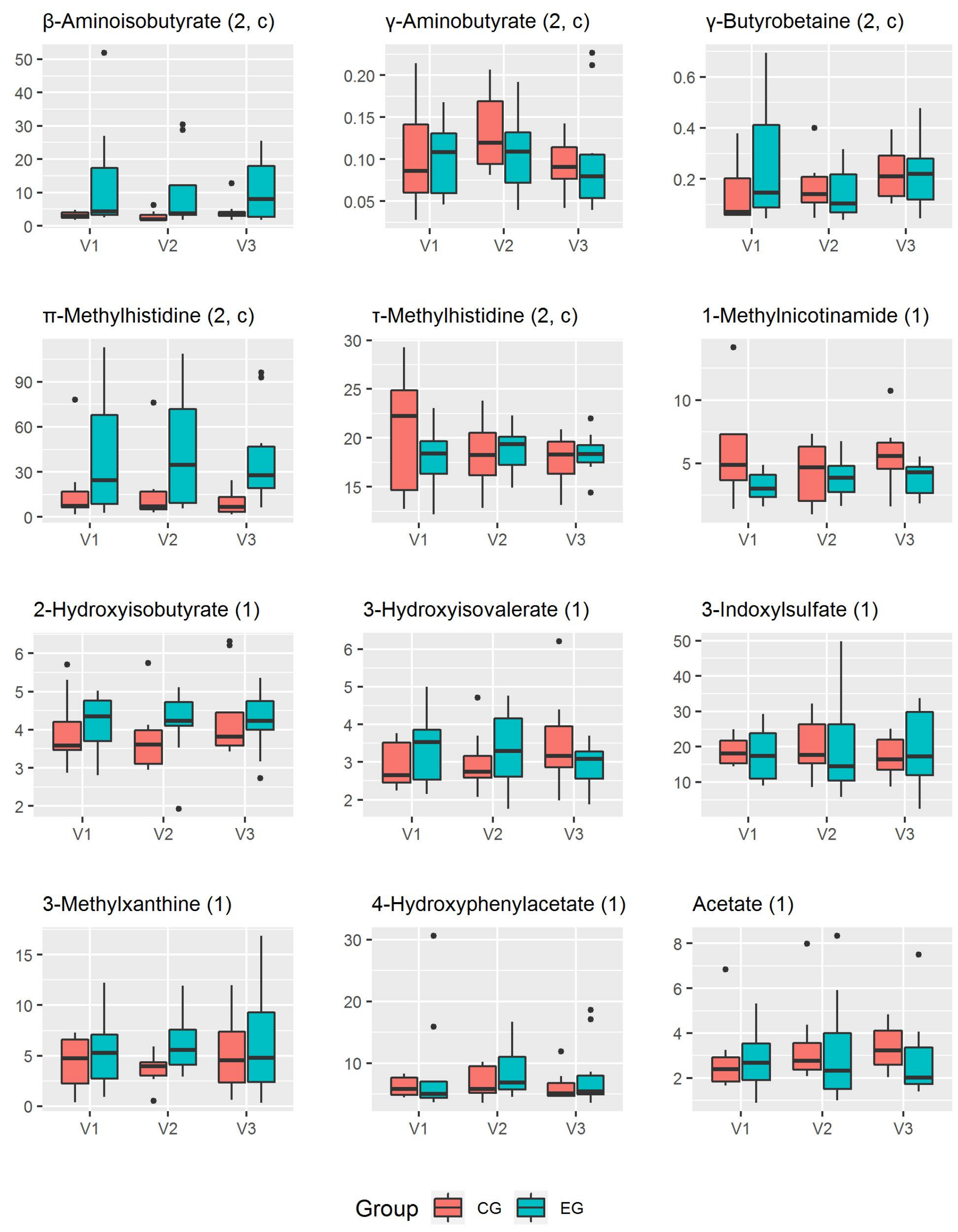

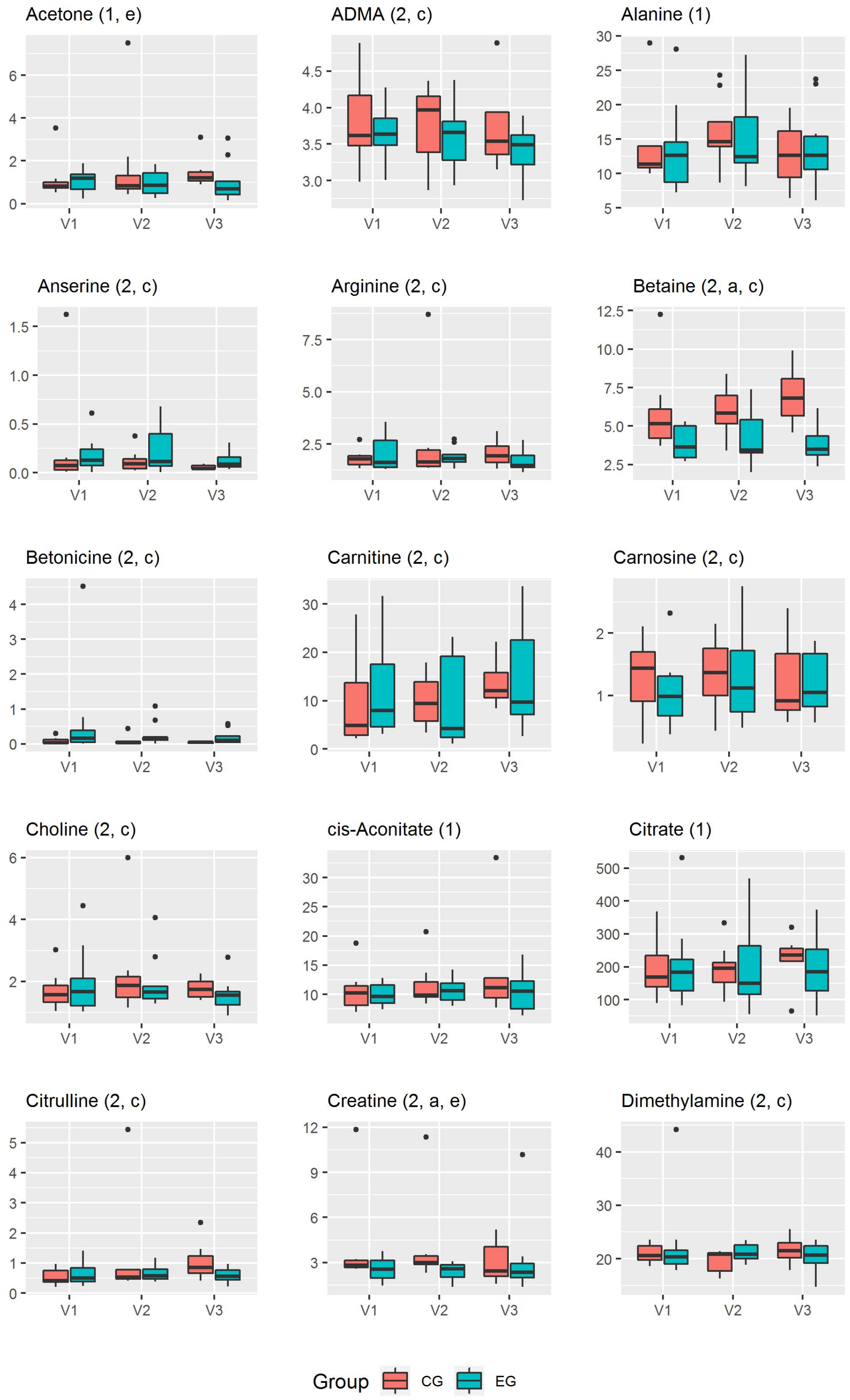
Acetone (1, e)
ADMA (2, c)
Alanine (1)
Anserine (2, c)
Arginine (2, c)
Betaine (2, a, c)
Betonicine (2, c)
Carnitine (2, c)
Carnosine (2, c)
Choline (2, c)
cis-Aconitate (1)
Citrate (1)
Citrulline (2, c)
Creatine (2, a, e)
Dimethylamine (2, c)
V1
V2
V3
Group
CG
EG

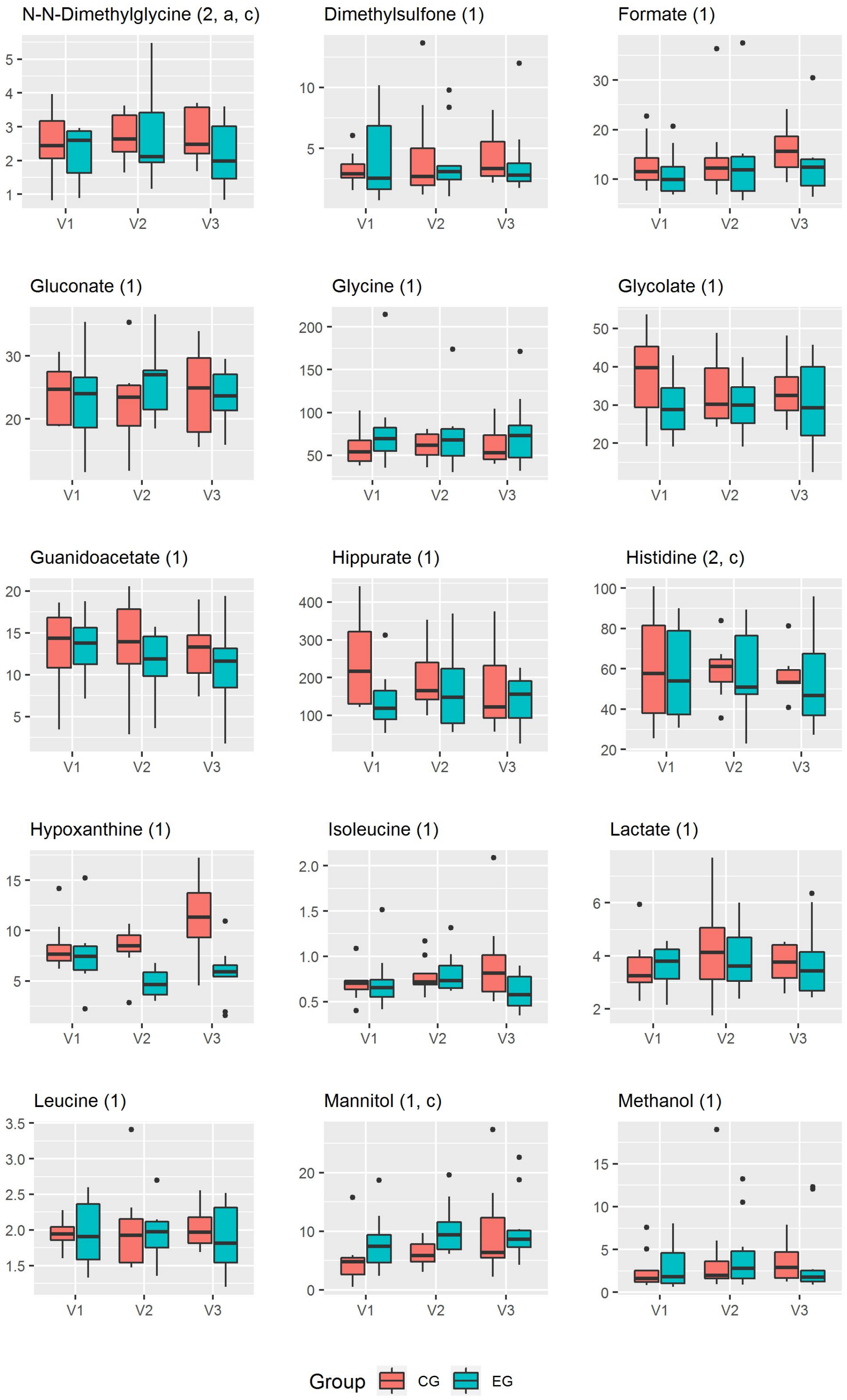

N-N-Dimethylglycine (2, a, c)
Dimethylsulfone (1)
Formate (1)
Gluconate (1)
Glycine (1)
Glycolate (1)
Guanidoacetate (1)
Hippurate (1)
Histidine (2, c)
Hypoxanthine (1)
Isoleucine (1)
Lactate (1)
Leucine (1)
Mannitol (1, c)
Methanol (1)
V1
V2
V3
Group
CG
EG

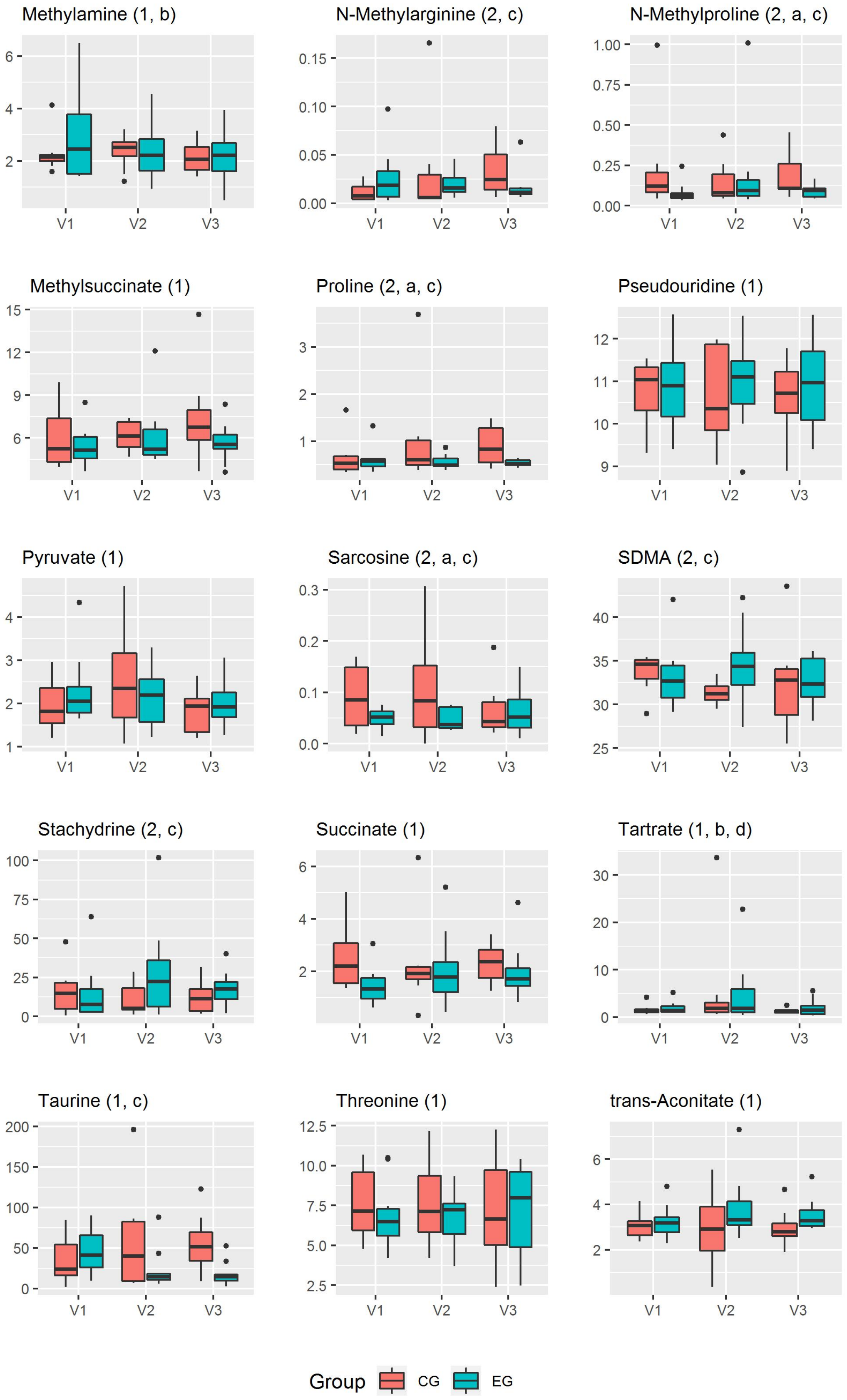
Methylamine (1, b)
N-Methylarginine (2, c)
N-Methylproline (2, a, c)
Methylsuccinate (1)
Proline (2, a, c)
Pseudouridine (1)
Pyruvate (1)
Sarcosine (2, a, c)
SDMA (2, c)
Stachydrine (2, c)
Succinate (1)
Tartrate (1, b, d)
Taurine (1, c)
Threonine (1)
trans-Aconitate (1)
V1
V2
V3
Group
CG
EG

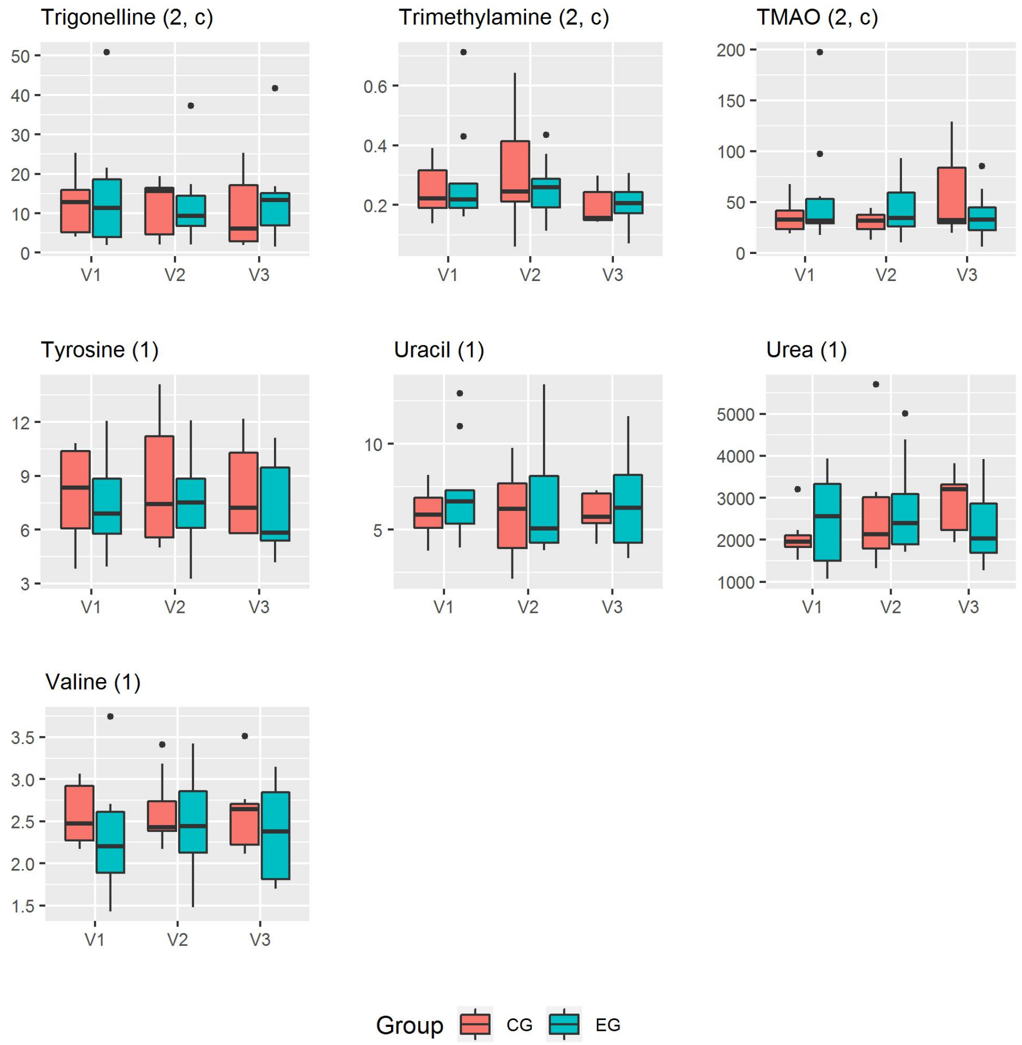

ADMA: asymmetric dimethylarginine, CG: control group, EG: experimental group, SDMA: symmetric dimethylarginine, TMAO: trimethylamine-N-oxide, V1: Visit 1 (day before the training started), V2: Visit 2 (day after the last training session), V3: Visit 3 (after four days of recovery); 1 NMR-detected, 2 LC-MS-detected;

n=10 (EG) and n=8 (CG), except: a) n=9 for EG, b) n=9 for EG only at V2, c) n=7 for CG, d) n=7 for CG only at V2, e) n=7 for CG only at V3.
