# Supplementary material for: High-Intensity Interval Training Decreases Resting Urinary Hypoxanthine Concentration in Young Active Men—A Metabolomic Approach
Source: Metabolites. 2019 Jul 10;9(7):137. doi: 10.3390/metabo9070137 (PMC6680906; doi:10.3390/metabo9070137)

Figure S2: Comparison of LC-MS and NMR metabolite data

linear regression	β-Aminoisobutyrate	Betaine	Carnitine	Creatine	Dimethylamine	N,N-Dimethylglycine	Histidine	TMAO	Trigonelline
r	0.99	0.95	1.00	0.93	0.97	0.98	0.99	1.00	0.99
r^2	0.97	0.90	0.99	0.87	0.94	0.96	0.98	0.99	0.99
intercept	-13.38	-1.07	-1.14	-6.46	21.12	-5.12	50.50	-17.85	-3.21
slope	0.99	0.94	1.18	0.81	0.64	1.25	1.11	1.46	0.94

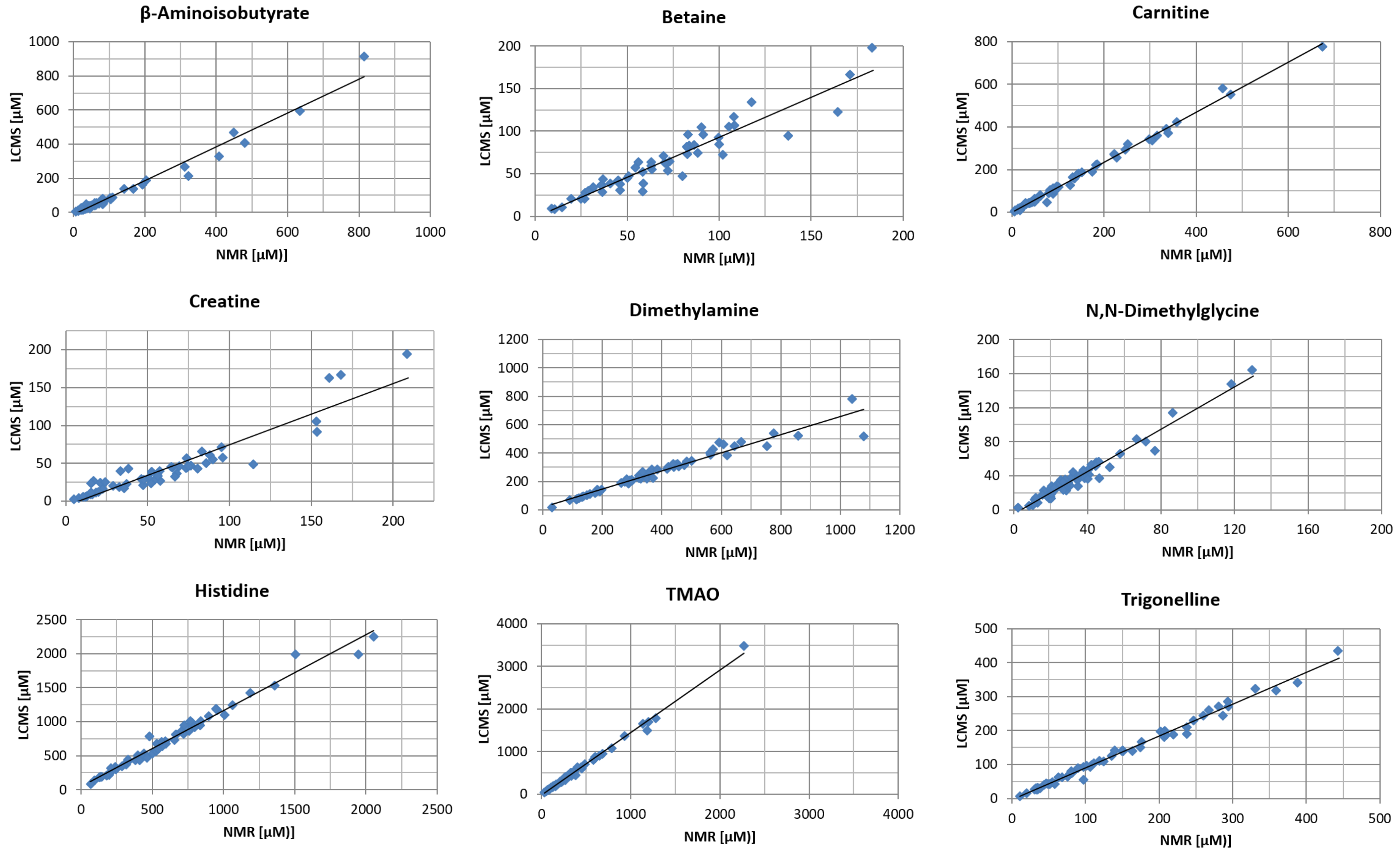

Supplement: Supplementary file 1 [file metabolites-09-00137-s001.zip › Figure_S2_Comparison of LC-MS and NMR metabolite data.pdf]
