# Supplementary material for: High-Intensity Interval Training Decreases Resting Urinary Hypoxanthine Concentration in Young Active Men—A Metabolomic Approach
Source: Metabolites. 2019 Jul 10;9(7):137. doi: 10.3390/metabo9070137 (PMC6680906; doi:10.3390/metabo9070137)
Supplement: Supplementary file 1 [file metabolites-09-00137-s001.zip › Table S4_LLOQ of LC-MS data.pdf]

Table S4: LLOQ of LC-MS data

Metabolites	LLOQ [µM] (S/N 10:1, CV <30%, Bias <30%)	Number of values below LLOQ (total sample size = 51)
β-Aminoisobutyrate	1.28	0
γ-Aminobutyrate	0.85	22
γ -Butyrobetaine	0.50	9
π-Methylhistidine	10.06	2
τ-Methylhistidine	10.81	0
ADMA	3.59	0
Anserine	24.02	51
Arginine	4.33	0
Betaine	1.08	0
Betonicine	1.00	31
Carnitine	2.35	0
Carnosine	5.31	11
Choline	1.23	0
Citrulline	5.98	21
Creatine	10.28	7
Dimethylamine	12.94	0
N,N-Dimethylglycine	2.09	0
Histidine	13.97	0
Hydroxyproline	10.29	0
N,N-Methylarginine	0.73	51
N,N-Methylproline	0.35	2
Proline	2.43	1
Sarcosine	1.18	37
SDMA	19.42	0
Stachydrine	1.78	1
TMAO	29.85	0
Trigonelline	2.23	0
Trimethylamine	0.21	0
